# Platelet count to spleen thickness ratio is related to histologic severity of primary biliary cholangitis

**DOI:** 10.1097/MD.0000000000009843

**Published:** 2018-02-16

**Authors:** Zhongfeng Wang, Xu Liu, Hongqin Xu, Limei Qu, Dezhi Zhang, Pujun Gao

**Affiliations:** a Department of Hepatology; b Department of Pathology; c Department of Abdomen Ultrasound, the First Hospital of Jilin University, Jilin University, Xinmin Street, Changchun, China.

**Keywords:** noninvasive markers, platelet count, primary biliary cholangitis, spleen thickness

## Abstract

The aim of this study was to evaluate the ability of noninvasive markers to identify the histological severity of primary biliary cholangitis (PBC).

Fifty-eight treatment-naïve PBC patients who had undergone liver biopsy were enrolled in our study. The patients’ histological stages were based on the classifications of Ludwig and Scheuer. Aspartate aminotransferase-to-platelet ratio index (APRI), fibrosis index based on the 4 factors (FIB-4), red blood cell distribution width to platelet ratio (RPR), and platelet count to spleen thickness (PC/ST) ratio were calculated. Using the area under the receiver operating characteristic curve (AUROC) to evaluate the accuracy of different markers for predicting the histological severity.

Among the 58 treatment-naïve PBC patients, the patients of Scheuer stage I/II/III/IV were 17/25/11/5, respectively. PC/ST ratio (AUROC = 0.807) was superior to RPR (AUROC = 0.717), APRI (AUROC = 0.726), FIB-4 (AUROC = 0.722), and mean platelet volume (MPV) (AUROC = 0.671) in discriminating between stage I and stage ≥II. The AUROC of PC/ST ratio, RPR, APRI, FIB-4, and MPV were 0.939, 0.872, 0.816, 0.831 and 0.572, respectively, for Scheuer stage ≥III; 0.968, 0.795, 0.744, and 0.723, respectively for stage IV. The sensitivity and specificity of PC/ST ratio were 73.4%,79.1%; 81%,100%;88.7%,100% for detection of Scheuer stage ≥ II, Scheuer stage ≥ III and Scheuer stage IV, respectively.

Our study findings indicated that compared with previous noninvasive test PRP, APRI, FIB-4 and MPV, PC/ST ratio shows the most accurate for distinguish the histologic severity of PBC patients.

## 1 Introduction

Primary biliary cholangitis (PBC) is a progressive autoimmune cholestatic liver disease that is characterized by the detection of the highly specific serum antimitochondrial antibody (AMA) and the destruction of small- and medium-bile ducts, which results in chronic cholestasis, portal inflammation, fibrosis, cirrhosis, and eventually liver failure or liver cancer.^[1]^ Diagnosis of PBC is based on a characteristic combination of biochemical, immunological, and histological features plus the exclusion of other causes of liver disease.^[2,3]^

Liver biopsy remains the gold standard for assessment of disease severity, but biopsies are limited by sampling error, invasiveness, cost, poor compliance, and contraindications, particularly in the follow-up.^[4]^ However, evaluation of the histologic severity provides valuable information with respect to the stage of the disease and aids in monitoring the response to treatment, which in turn provides information related to the prognosis.^[5,6]^ Hence, the need to establish noninvasive methods to replace liver biopsy in the assessment of histological severity has become urgent because noninvasive approaches are associated with lower risk and are often less expensive than liver biopsy.

Noninvasive tests, Mac-2-binding protein (M2BP), RPR, MPV, APRI, and FIB-4 have been proposed to detect the histological severity of PBC.^[7–10]^ Nevertheless, seldom studies focused on the ultrasound, especially spleen data. Accordingly, our objective in the present study was to evaluate the ability of noninvasive markers to identify the histological severity of PBC.

## 2 Materials and methods

This retrospective study was performed in the First Hospital of Jilin University between January 2010 and June 2016. The diagnosis of PBC was established in accordance with the EASL 2009 guidelines.^[3]^ The exclusion criteria were: (a) PBC-AIH overlap syndrome or some other coexisting liver diseases. (b) Biopsy sample length <15 mm and/or including <6 portal tracts, and incomplete clinical and/or laboratory data. At last, 58 treatment-naïve PBC patients were included in the final analysis. All study subjects gave written informed consent for liver biopsy. The study was conducted in accordance with the Declaration of Helsinki and approved by the Ethics Committee of the First Hospital of Jilin University.

Demographical, abdominal ultrasound (US), clinical, and laboratory data within the last 1 week before liver biopsy were collected and registered in a form by an uninformed clinician to prevent bias.

A 16G Tru-Cut needle was applied in color Doppler ultrasound-guided liver biopsy. The specimens were fixed in buffered formalin, embedded in paraffin and stained with hematoxylin and eosin (H&E), Masson^'^s. The pathological diagnosis of each liver biopsy tissue was determined from Ludwig's and Scheuer's classifications after a double-blind inspection by 2 specialists in the Pathological Diagnostic Center at the First Hospital of Jilin University. Disease stage can be categorized into 4 stages according to this histological staging system. Florid duct lesions are seen in stage I, ductular proliferation in stage II, scarring in stage III, and finally cirrhosis in stage IV. Measurement method of spleen thickness: Ultrasonic probe locates in left intercostal skew, the minimum distance between the spleen and the opposite convex surface of the spleen is measured at the smallest cross section of the spleen. So is more easy and accurate associated with spleen diameter. Also it is an innovation in our study.

### 2.1 Formulae

 
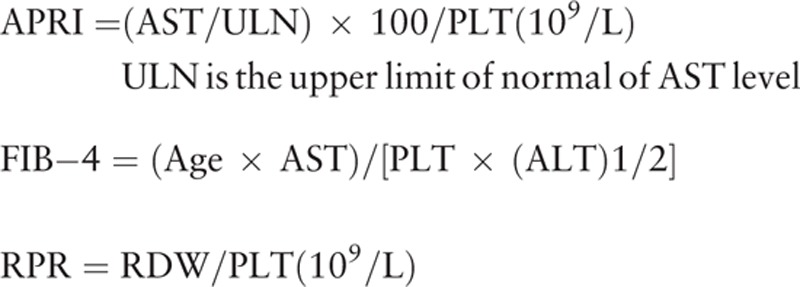


PC/ST ratio = platelet count (N/mm^3^)/spleen thickness (mm)

### 2.2 Statistical analysis

Continuous variables were shown as mean (25th/75th percentile or 95% confidence interval). Categorical variables were displayed as numbers and percentages. The Kruskal–Wallis nonparametric test was used for multiple comparisons. Student *t* test and Mann–Whitney nonparametric *U* test were used for comparison of continuous variables between 2 groups, as appropriate. The diagnostic value of APRI, FIB-4, RPR, MPV, and PC/ST ratio were assessed using the area under the receiver-operating curve (AUROC), the sensitivity and specificity to detect PBC stage were calculated by the optimal cut-off value of each test, respectively. The positive predictive values (PPV) and negative predictive values (NPV) for the appropriate cut-offs were calculated. All statistical analyses were performed in SPSS 20.0 (SPSS Inc, Chicago, IL). All *P* values given are 2-sided and a *P* value < .05 is statistically significant.

## 3 Results

Between January 2010 and June 2016, 112 patients with PBC were admitted to the Department of Hepatology at the First Hospital of Jilin University. A total of 54 patients were excluded from the study for the following reasons: 9 patients had AIH-PBC, 14 had coexisting liver diseases, 6 had liver tissue specimens inadequate for staging of fibrosis, 31 had incomplete laboratory or ultrasound data. Overall, 58 patients with an established diagnosis of PBC were included in the study. The baseline characteristics of patients are presented in Table 1. There were 51 (87.9%) women and 7 men, with a median age of 53.3 years (interquartile range, IQR 49.5–59). The percentage of patients in Scheuer stage I, II, III, and IV hepatic fibrosis were 17 (29.3%), 25 (43.1%), 11 (19.0%), and 5 (8.6%), respectively. In addition, the raw data about PC/ST ratio, RPR, APRI, FIB-4, and MPV of patients depending on the histological staging have been showed in Table 2.

**Table 1:**
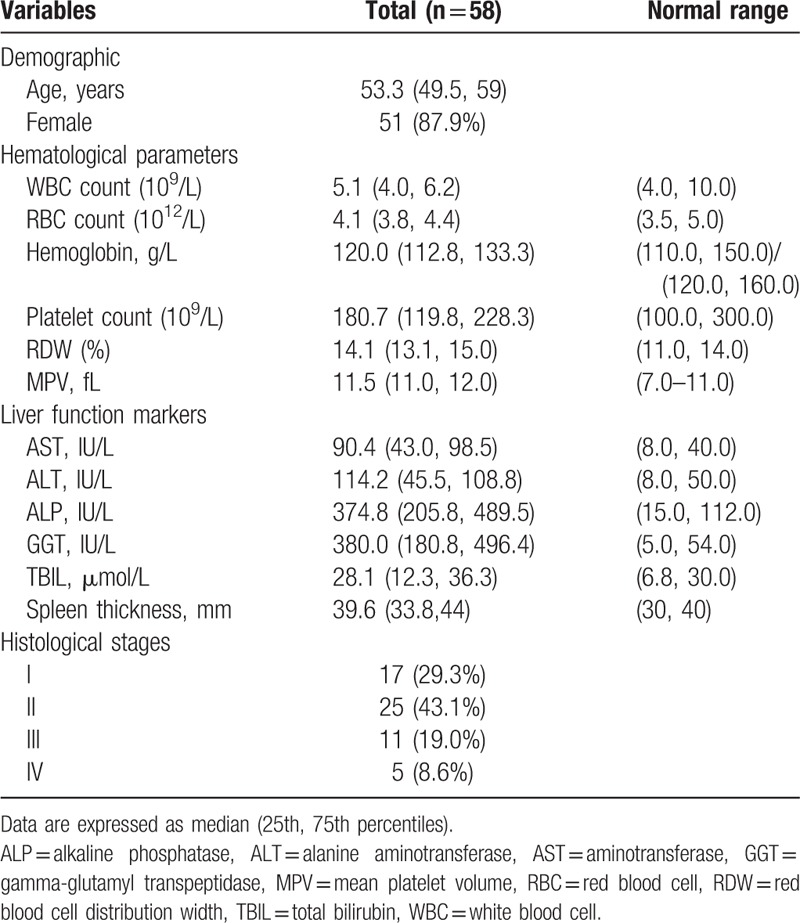
Baseline characteristics of study population.

**Table 2:**
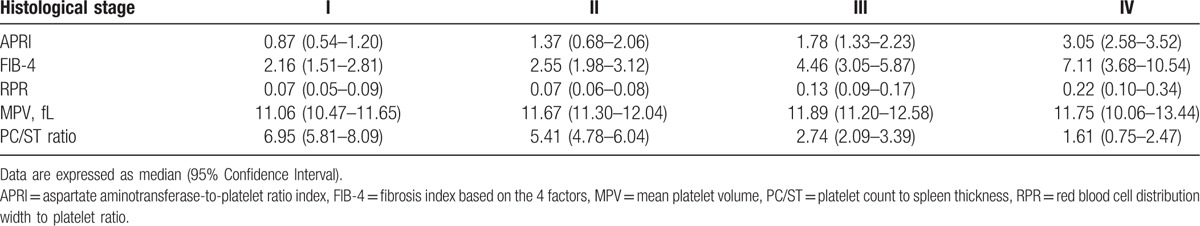
Data about PC/ST ratio, RPR, APRI, FIB-4, and MPV depending on the histological staging.

**Figure 1:**
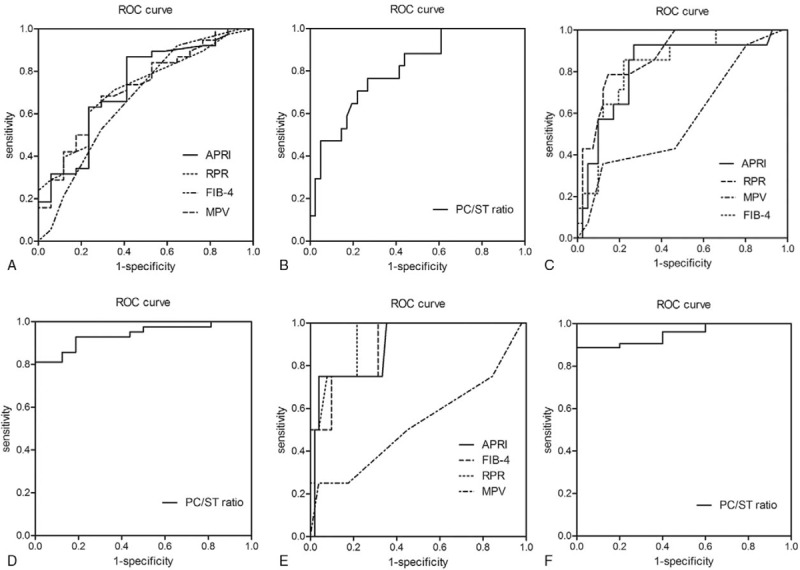
Receiver operator characteristics curves for RPR, APRI, FIB-4, FIB-4, and PC/ST ratio for the diagnosis of different Scheuer stages: (A and B) Scheuer stages ≥II; (C and D) Scheuer stages ≥III; and (E and F) Scheuer stages IV. APRI = aspartate aminotransferase-to-platelet ratio index, FIB-4 = fibrosis index based on the 4 factors, PC/ST = platelet count to spleen thickness, RPR = red blood cell distribution width to platelet ratio.

According to the analysis of AUROC, the PC/ST ratio (AUROC = 0.807; 95% confidence interval, CI 0.688–0.926, *P < *.001) was superior to RPR (AUROC = 0.717; 95% CI 0.576–0.858, *P = *.006), APRI (AUROC = 0.0.726; 95% CI 0.577–0.875, *P = *.004), FIB-4 (AUROC = 0.722; 95% CI 0.578,0.866, *P = *.006) and MPV (AUROC = 0.671; 95% CI 0.506–0.836, *P = *.036) in distinguishing between Stage I and Stage ≥II in patients with PBC (Fig. 1A and B). For predicting Scheuer stage ≥II, the optimal cut-off value of PC/ST ratio is 5.38, the sensitivity and specificity were 76.5% and 73.2%, respectively.

For predicting Scheuer stage ≥III, PC/ST ratio also shows the highest AUROC (0.939; 95% CI 0.881–0.997, *P < *.001), the sensitivity and specificity were 81% and 100%, respectively. However, the AUROC of RPR, APRI, FIB-4, MPV were 0.872, 0.816, 0.83,1 and 0.572, respectively (Fig. 1C and D). Meanwhile, the optimal cut-off value of PC/ST ratio is 4.21. For predicting Stage IV, the AUROC of PC/ST ratio (0.951, 95% CI 0.000–1.000, *P = *.001) was significantly better than that of PPR (0.926, 95% CI 0.000–1.000, *P = *.002), APRI (0.895, 95% CI 0.000–1.000, *P = *.003), FIB-4 (0.897, 95% CI 0.000–1.000, *P = *.004) and MPV (0.527, 95% CI 0.181–0.872, *P = *.825), the optimal cut-off value of PC/ST ratio is 2.59, with a sensitivity and specificity of 88.7% and 100%, respectively (Fig. 1E and F).

## 4 Discussion

In EASL guidelines, the diagnosis of PBC is made with 2 of the followings: (a) antimitochondrial antibody (AMA) or antimitochondrial M2 antibody (AMA-M2) positive; (b) unexplained elevated alkaline phosphatase (ALP) 1.5 times the normal upper value for over 24 weeks; (c) histologic evidence of nonsuppurative destructive cholangitis, and destruction of interlobular bile ducts.^[3]^ Recently, with the development of testing methods, most PBC patients are diagnosed with increased ALP levels and AMA positivity, hence, liver biopsy became not that urgent in the diagnosis of PBC. On one hand, most patients with PBC show slow progression and respond to medical treatment in the early stages, on the other hand, the presence of cirrhosis (stage IV) is associated with a poor prognosis and these patients are at an increased risk of developing cirrhosis-related complications. Hence, histological evaluation is still useful in the pathological assessment of PBC, and to predict prognosis, but biopsies are limited by sampling error, invasiveness, cost, poor compliance, and contraindications, particularly in the follow-up. Nowadays, increasingly more noninvasive markers or models have been proposed to predict the histologic severity of liver fibrosis, especially for virus hepatitis, for example, APRI has been recommended as the preferred noninvasive test to assess for the presence of cirrhosis (APRI score > 2 in adults) in resource-limited settings.^[11]^ However, studies investigating the relationship between noninvasive tests and histological stages of fibrosis are limited in PBC.

In the last decade, several studies have been carried out to evaluate liver fibrosis and noninvasive methods in PBC.^[8,9,12,13]^ To our knowledge, this study is the first to evaluate noninvasive test to predict histological severity of PBC with spleen diameters. In this study, we assessed the diagnostic performance of five noninvasive parameters for the histological severity of PBC. There was a statistically significant moderate positive correlation between RPR, APRI, and FIB-4 values and Ludwig^'^s stage, as well as a strong negative correlation between the PC/ST ratio and Ludwig^'^s stage on liver biopsy. We found that the PC/ST ratio is superior to RPR, APRI, FIB-4, and MPV in predicting of histological severity in patients with PBC. The PC/ST ratio, including 2 indicators of portal hypertension was initially designed to predict esophageal varices in patients with liver cirrhosis.^[14,15]^ The noninvasive diagnosis of liver fibrosis in our study was stand by the fact that both spleen size and platelet count were independently and significantly associated with extent of liver fibrosis in multivariate analysis. According to our study, PC/ST ratio performed better in identifying Scheuer stage≥II, stage≥III, and stage IV than other noninvasive markers, RPR, APRI, FIB-4, and MPV.

To our knowledge, there are few studies evaluating noninvasive markers of histological severity in patients with PBC. Wang et al^[8]^ found RDW and RPR were related to histologic severity of PBC, and its AUROC is better than APRI and FIB-4. Alempijevic et al^[16]^ found a statistically significant correlation between disease stage and AST levels, APRI. Trivedi et al^[17]^ showed that APRI is associated with the optimising risk stratification in PBC and can predict patients^’^ outcome independent of ursodeoxycholic acid response. Olmeza and colleagues^[13]^ evaluated 40 patients found that there were statistically significant differences in APRI, FIB-4 scores between the groups with early (Scheuer stages I and II) and advanced stages (Scheuer stages III and IV) of disease. It is found that there was no difference among patients between early stage and advanced stage disease in terms of APRI scores, but MPV values were different between groups. In our study, we enrolled treatment-naïve PBC patients and found that RPR, APRI, FIB-4 showed high specificity and sensitivity for detection of Scheuer stage ≥II, ≥III and stage IV, respectively, with AUROC more than 0.8. However, we did not detect significant increases in MPV levels between different stages, may just because their sample size of 39 patients was small. In addition, the studies before are just focus on distinguish the early stage stages and advanced stages with noninvasive markers, our study paid attention to the four stages and find the optimal cut-off values to predict different stages.

This study has several limitations. Firstly, the patients in our study were part of a single-center study and retrospectively enrolled, and our models have not been validated as yet with large prospective studies. Secondly, because of the lack of data, we investigate the spleen thickness (ST) other than spleen diameters (SD), which has been promoted before to identify the severity of esophagus varices. Thirdly, the sample size of our study is relatively small, due to the incomplete data of blood test and ultrasound, we select the patients with complete data.

In conclusion, our study findings indicated that PRP, APRI, FIB-4, and PC/ST ratio could provide useful information for the prediction of histologic severity in PBC patients, which could aid in reducing the need for liver biopsy. Meanwhile, PC/ST ratio shows the highest AUROC for prediction different stages than other noninvasive markers with reasonable sensitivity and specificity. As a result, we can use the optimal cut-off values of PC/ST for the diagnosis of disease severity of PBC patients, also the treatment effect prediction and follow up.
